# Retention in Care of HIV-Infected Children from HIV Test to Start of Antiretroviral Therapy: Systematic Review

**DOI:** 10.1371/journal.pone.0056446

**Published:** 2013-02-20

**Authors:** Catrina Mugglin, Gilles Wandeler, Janne Estill, Matthias Egger, Nicole Bender, Mary-Ann Davies, Olivia Keiser

**Affiliations:** 1 Division of International and Environmental Health, Institute of Social and Preventive Medicine (ISPM), University of Bern, Switzerland; 2 Department of Infectious Diseases, Bern University Hospital, Bern, Switzerland; 3 School of Public Health and Family Medicine, University of Cape Town, South Africa; London School of Hygiene and Tropical Medicine, United Kingdom

## Abstract

**Background:**

In adults it is well documented that there are substantial losses to the programme between HIV testing and start of antiretroviral therapy (ART). The magnitude and reasons for loss to follow-up and death between HIV diagnosis and start of ART in children are not well defined.

**Methods:**

We searched the PubMed and EMBASE databases for studies on children followed between HIV diagnosis and start of ART in low-income settings. We examined the proportion of children with a CD4 cell count/percentage after after being diagnosed with HIV infection, the number of treatment-eligible children starting ART and predictors of loss to programme. Data were extracted in duplicate.

**Results:**

Eight studies from sub-Saharan Africa and two studies from Asia with a total of 10,741 children were included. Median age ranged from 2.2 to 6.5 years. Between 78.0 and 97.0% of HIV-infected children subsequently had a CD4 cell count/percentage measured, 63.2 to 90.7% of children with an eligibility assessment met the eligibility criteria for the particular setting and time and 39.5 to 99.4% of the eligible children started ART. Three studies reported an association between low CD4 count/percentage and ART initiation while no association was reported for gender. Only two studies reported on pre-ART mortality and found rates of 13 and 6 per 100 person-years.

**Conclusion:**

Most children who presented for HIV care met eligibility criteria for ART. There is an urgent need for strategies to improve the access to and retention to care of HIV-infected children in resource-limited settings.

## Introduction

In 2010 worldwide about 3.4 million children younger than 15 years were HIV infected, of whom over 90% lived in sub-Saharan Africa [1]. In low-income countries, more than half of vertically-infected children die before the age of two years if they remain untreated [2]. Although early antiretroviral therapy (ART) has been shown to dramatically reduce early mortality and progression of HIV [3], the estimated coverage of ART in low and middle income countries is still much lower in children than in adults: in 2010 23% of the children in need of therapy received ART compared to 51% of adults [1]. However, the reasons for poor uptake of HIV testing and low therapy coverage in children are poorly understood.

A major challenge of health care programmes in the context of the rapid scale-up of ART is to retain patients in care after they tested positive for HIV. Retention is particularly poor in patients not yet eligible for ART. A recent systematic review in adults showed that only 59% of HIV positive patients had a CD4 count to determine treatment eligibility and that only 68% of ART eligible patients started ART [4]. Higher pre-ART retention in adults was associated with the availability of a point-of-care CD4 test [5], [6] and a better health status of the patient [7]. There are few data on loss to programme (mortality, loss to follow-up and transfer out) between HIV testing and start of ART in children. Good retention in care in children may be associated with similar factors as in adults but will in addition depend on the caregiver.

We performed a systematic review to estimate the magnitude and reasons for loss to programme between HIV testing and start of ART in HIV infected children in low-income settings.

## Materials and Methods

### Data sources

We searched the PubMed and EMBASE bibliographic databases on August 9, 2011. We limited the search to English-language publications which reported on patients in low-income settings. We further limited the search to studies published from 2002 onwards because the scale-up of ART in resource-limited settings (as defined by the World Bank classification) happened after 2002 [8], [9]. We used both free text and Medical Subject Headings (MeSH) and used a combination of the following words and their variations: ‘antiretroviral agents’, ‘therapeutic use’, ‘pre treatment’, ‘pre-ART’, ‘prior to treatment’, ‘eligibility’, ‘loss to care’ and ‘loss to follow-up’. We examined the references of all included studies. Further details of the search strategy are shown in the Appendix S1.

### Study selection

We included all studies that reported on numbers of children followed between HIV diagnosis and start of ART, including studies that did not cover the entire time period. We excluded studies on adults and on the prevention of mother-to-child transmission (PMTCT). We also excluded qualitative studies, data from clinical trials and reports from national programmes (as there was a risk of duplicated data and lack of detailed information). Articles were excluded if they reported on the same study population and time period as another article that was more complete. Two reviewers (C.M., O.K.) assessed the eligibility of articles and abstracts. Discrepancies were resolved by consensus between the two reviewers.

### Data extraction and analysis

We extracted the data of each publication in duplicate using a standardised data extraction sheet. The following information was extracted for each study: inclusion criteria, characteristics of the programme (setting, location, country), characteristics of the children (age, gender, CD4 cell counts or percentages at different time points), eligibility criteria for ART initiation and methods for tracing children lost to follow-up. In addition we extracted the number of children alive or lost to programme (i.e. lost to follow-up, transferred-out or died) during different time periods. The following time points were of interest: HIV testing, CD4 testing or clinical staging with eligibility assessment for ART, becoming eligible for ART and start of ART. We also assessed the number of children staying in pre-ART care and overall losses to programme and mortality before ART initiation or during pre-ART care. Finally we extracted predictors for loss to programme, mortality and loss to follow-up between HIV testing and CD4 testing and between meeting eligibility criteria for ART and ART initiation. We recorded if there was a positive or negative statistically significant association (p<0.05) or if there was no statistically significant association (p≥0.05). Discrepancies were resolved by consensus. Data were entered into an EpiData database (version 3.1). We calculated the percentage of children completing each time step and displayed the results as forest plots. Data were analysed using STATA version 11.2 (StataCorp, Texas, USA).

## Results

### Study characteristics

**Figure 1:**
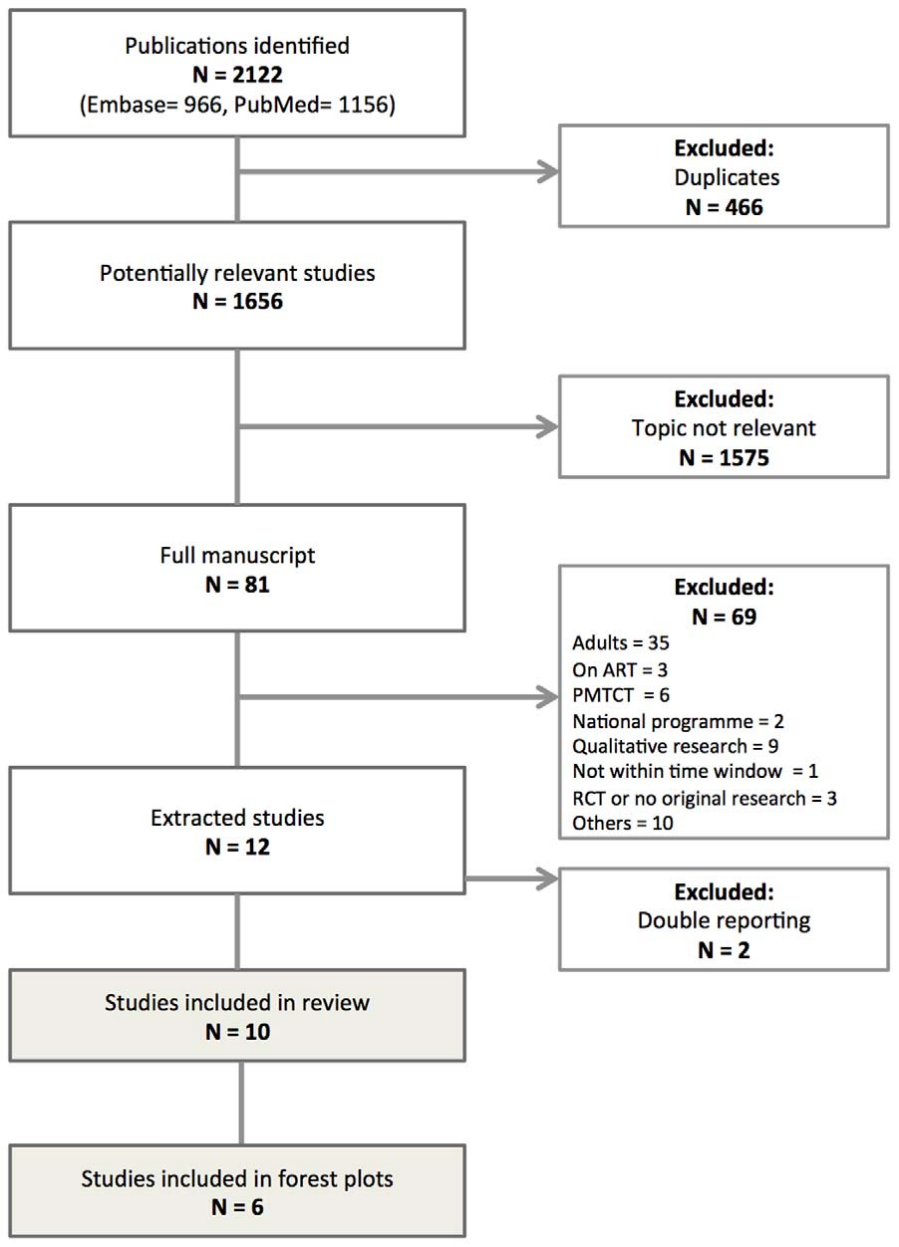
Identification and selection of studies. ART: Antiretroviral therapy; RCT: Randomized controlled trial; PMTCT: Prevention of mother to child transmission.

We identified 1,656 potentially relevant articles (Figure 1). Ten studies were included in the systematic review (Table 1): eight studies from sub-Saharan Africa (one study each from Côte d'Ivoire, the Democratic Republic of Congo, Ethiopia, Lesotho, Malawi, South Africa, Zambia and Kenya); one study from India; one study from Cambodia. The studies contributed data on 10,741 children and included all children who attended routine clinics during a specified time period, except for one study [10], in which a random sample of children was analyzed. Most of the studies were multi-site (n = 8); two studies were conducted at one site. The majority of studies reported on programmes in urban clinics where patients were seen by medical doctors. Table 1 shows a detailed description of the programmes studied, including the level of care delivery and the funding sources for the study. The median age of the children ranged from 2.2 to 6.5 years. Median or mean CD4 cell count at presentation ranged from 385 to 622 cells/µl, and median CD4 percentage from 14.5% to 16%. A detailed listing of CD4 cell measurement at presentation and corresponding age group in the study can be found in Table 1.

**Table 1:** Characteristics of studies included in review, first CD4 cell count measurement after HIV diagnosis and mortality rates prior to initiation of antiretroviral therapy (ART).

First author, year	Location	Setting	Facilities	Funding	Care delivery by	Study period	Nr children	Age (yrs)	HIV diagnosis	Eligibility for ART*	Median baseline CD4**
Anaky, 2011 [11]	Cote d'Ivoire	Semi-urban, urban	17 urban and 2 semi-urban clinics in Abidjan	PEPFAR	trained health care workers	2004 - 2007	1766	median (IQR):4.5 (1.8 - 8.2)	>18 mths rapid assay,<18 mths RNA PCR	CD4% <25%: age <12mths,CD4% <20%: 12-35mths,CD4% <15%: ≥36mths	A: 487P: 15.6
Berhan, 2011 [10]	Ethiopia	n.r.	7 referral public hospitals, 2 are located in the capital	n.r.	general practitioner physicians, pediatricians	2008 - 2009	1163	mean (SD):4.9 (3.2)range: 1 mo – 14 yrs	n.r.	n.r.	n.r. n.r.
Edmonds, 2011 [12]	DRC	urban	2 hospitals in Kinshasa	n.r.	comprehensive HIV care and treatment programme	2007 - 2010	790	median (IQR):5.9 (2.7 - 9.8)	<18 mths DNA PCR,>18 mths serological testing or HIV viral load	WHO guidelines 2006/2010, national guidelines	P: 15
Feucht, 2007 [19]	South Africa	urban	Regional state hospital, pediatric ART clinic	n.r.	n.r.	2004	276	mean (range):4.3 (0.1 - 13)	n.r.	South African guidelines 2003	A: mean: 622P: mean 15.3
Leyenaar, 2010 [13]	Lesotho	urban	Pediatric HIV/AIDS care and treatment facility	Bristol-Myers Squibb, Baylor college	Nurses, social workers, physicians	2005 - 2007	567	median (range):2.2 (0 - 15.5)	n.r.	National guidelines (based on WHO 2006)	P: mean 15
McGuire, 2010 [14]	Malawi	rural	1 district hospital, 10 health centers	Médecins sans Frontières	n.r.	2001 - 2007	107	n.r.	n.r.	n.r.	n.r.
Nyandiko, 2009 [17]	Kenya	rural, urban	1 urban referral clinic, 17 outpatient services	USAID-AMPATH	Paediatricians, medical and clinical officers	2002 - 2008	4017	median (range):4.5 (0 - 14.2)	<18 mths DNA PCR,>18 mths 2 parallel ELISAs	CD4% <15%: <6 yrs,CD4 <200 cells/µl: >6 yrs	A: 484P: 16.0
Raguenaud, 2009 [16]	Cambodia	rural	2 hospitals, 1 pediatric clinic, 1 referral hospital	n.r.	Doctor based clinical care, follow up by multidisciplinary team	2002 - 2008	1168	n.r.	<18 months RT PCR since 2006	CD4% <15%: 36-59mths,CD4% <20%: 12-35mths,CD4 <200 cells/µl: ≥5 yrs WHO stage 3/ 4	A: 410P: 14.5
Seth, 2011 [18] ***	India	urban	1 tertiary teaching hospital. New Dehli	n.r.	n.r.	2006 - 2010	24	n.r.	<18 months DNA PCR,>18 months reactive HIV serology	n.r.	n.r.
Sutcliffe, 2010 [15]	Zambia	rural, urban	1 urban clinic: in a low income community in Lusaka, 2 rural clinics	Rural hospitals (churches, urban facility (Ministry of Health)	n.r.	2004 - 2008	863	median (IQR):urban: 6.5 (3.2 - 9.9),rural: 3.4 (1.8 - 7.4)	n.r.	WHO 2003/2006, national guidelines	A: urban: 385, rural: 572;P: n.r.

n.r. not reported.

* Immunological and clinical eligibility criteria for ART initiation were the following: WHO 2003 guidelines [33]: all children if WHO paediatric stage III. WHO paediatric stage I (only when CD4 count available) or paediatric stage II: <18 months: CD4 percentage <20%. ≥18 months: CD4 percentage <15%. WHO 2006 guidelines [20]: all children if WHO stage 3 or 4 (there are specific rules for WHO stage 3 in case of co-infections). WHO stage 1 or 2 (total lymphocyte counts are used in sites where CD4 values cannot be determined): <1 year: CD4 percentage <25% or absolute CD4 cell count <1500 cells/µl. 1 to <3 years: CD4 percentage <20% or absolute CD4 cell count <750 cells/µl. 3 to <5 years: CD4 percentage <15% or absolute CD4 cell count <350 cells/µl. ≥5 years: CD4 percentage <15% or absolute CD4 cell count <200 cells/µl. • WHO 2010 guidelines [21]: all children if <2 years or in WHO stage 3 or 4. WHO stage 1 or 2: 2 to <5 years: CD4 percentage ≤25% or absolute CD4 cell count ≤750 cells/µl. ≥5 years: ≤350 cells/µl.

** Absolute CD4 count A, percentage CD4 P.

*** Of 162 HIV exposed children, all 24 children who were diagnosed HIV positive were included.

### Mortality, loss to follow-up and transfer out

Deaths among patients lost to follow-up were ascertained in 5 studies by phone calls or home visits. Two studies reported on pre-ART mortality and found rates of 13.0 [11] and 6.0 [12] per 100 person-years (Table S1). A loss to follow-up rate and transfer-out rate before ART initiation were reported by one study [11] and were 50.3 and 2.9 per 100 person-years respectively. Four studies reported on percentages of children who died, were lost to follow-up/defaulted or transferred out before starting ART [13]–[16], and one study reported the proportion of patients lost to follow-up [17]. A small study of 24 children reported on the proportion of children who died, with no children lost to follow-up [18]. The estimates ranged between 3.2% and 45.8% for mortality, 0.0% and 37% for loss to follow-up and 3.4% and 4.6% for transfer out (Table S1). Since the person-time at risk was not reported these percentages are difficult to interpret.

### Pre ART cascade

Studies reporting at least one of the three steps (HIV diagnosis to CD4 cell determination, ART eligibility assessment to meeting eligibility criteria, meeting ART eligibility criteria to ART start) were included in this analysis.

### From HIV diagnosis to CD4 cell determination

**Figure 2:**
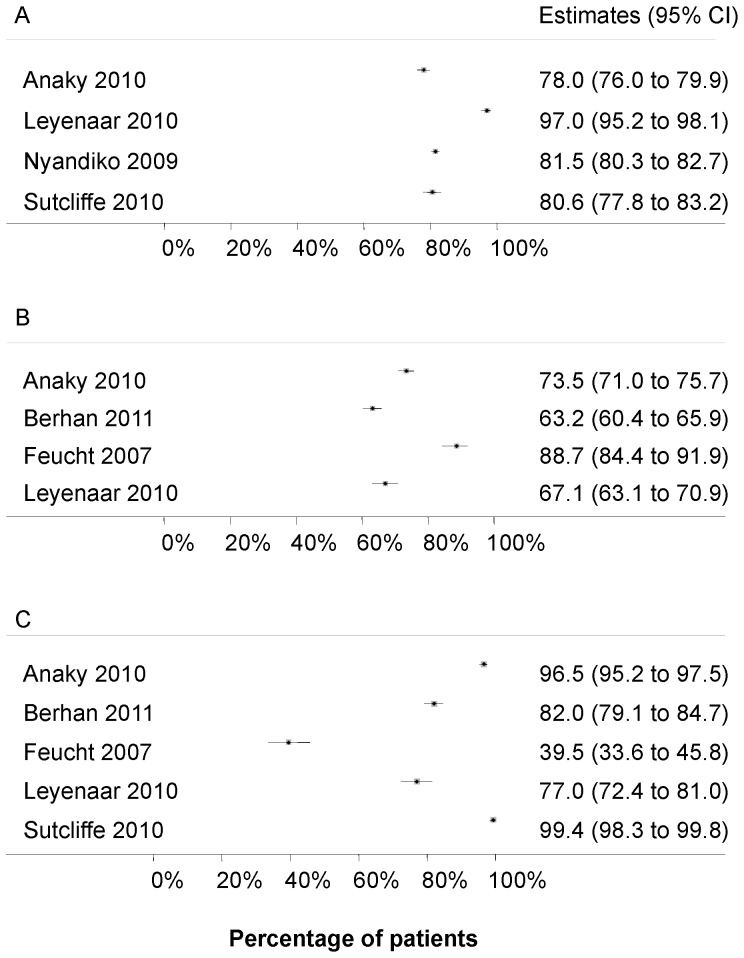
Forest plots – time from HIV diagnosis to start of antiretroviral therapy (ART). A): Percentage of HIV positive children with a CD4 cell count/percentage. B): Percentage of children with an eligibility assessement who meet eligibility criteria for ART. C): Percentage of ART eligible children starting ART.

Four studies [11], [13], [15], [17] reported on the step from HIV diagnosis to CD4 cell measurement. The percentage of children with a CD4 cell count ranged from 78.0% to 97.0% (Figure 2 , panel A).

### From assessment of eligibility to start of ART

Four studies [10], [11], [13], [19] reported on the period from assessment of eligibility to start of ART. The percentage of children diagnosed with HIV who had a CD4 cell count/percentage measured and were eligible for ART ranged from 63.2% to 88.7% (Figure 2 , panel B). Five studies [10], [11], [13], [15], [19] reported on the number of children starting ART after meeting eligibility criteria. Estimates ranged from 39.5% to 99.4% (Figure 2 , panel C).

### Predictors of mortality, loss to follow-up and ART initiation

Three studies reported on predictors for starting ART while no study reported on predictors for determining a CD4 cell count/percentage or of mortality. A low CD4 cell count was a strong predictor for ART initiation in all three studies [10], [12], [15]. Two studies reported no association between gender and ART initiation. Sutcliffe *et al* [15] reported that a long distance to the clinic was associated with loss to follow-up (in particular in children living in rural areas) and Edmonds *et al* [12] found that advanced clinical stage was associated with ART initiation.

## Discussion

Our systematic review included over 10,000 children from 10 low-income countries. In general, few data were available on the different outcomes and heterogeneity across studies was substantial. Our analysis showed that in low-income settings, 78% and 97% of HIV-infected children had a CD4 count/percentage measured and that the large majority of these patients met ART eligibility criteria at presentation. Among those who were assessed for ART eligibility, 63% to 91% of the children were already eligible for therapy, and of the eligible children 40% to 99% initiated ART. According to the WHO 2006 guidelines [20], most children in WHO stage 3 (with few exceptions) and all children in WHO stage 4 are eligible for ART irrespective of CD4 count or percentage, and children in WHO stage 1 and 2 are eligible if the CD4 percentage or absolute CD4 cell count is below an age-specific threshold. In the 2010 revision of the guidelines, WHO recommended that all children aged <2 years should start ART irrespective of the CD4 count, percentage or clinical stage [21]. Different eligibility criteria will influence the number of people deemed eligible for ART initiation while for children in advanced clinical stage it was not necessary to measure CD4 cells. Apart from two studies [12], [18] the follow-up ended before the new guidelines were published. Our study could thus not determine the impact of the change in guidelines on retention in care. Furthermore, operational and financial barriers were the source of differences between implementations of the new ART guidelines across countries. During the study period other major operational changes were made. This includes, for example scale-up of access to PCR HIV testing for children <18 months and use of paediatric syrups instead of soluble fixed dose combinations. This further limited comparison of treatment uptake overall, and for specific age groups and time periods in particular.

This systematic review has several other limitations: 1) The search strategy was limited to English-language publications from only two databases. 2) The generalizability of our findings was influenced by the geographic limitations of the search and the small number of studies. 3) Many studies did not explicitly report if the CD4 cell count used to determine treatment eligibility was the first measured CD4 cell count or if the measurement was taken during pre ART care. 4) The studies did not limit the analysis of predictors for ART initiation to ART eligible children only. 5) Only few studies reported on the outcomes of children not retained in care, as these were not traced systematically in most studies. 6) Finally, it was not always possible to distinguish between overall loss to programme and loss to follow-up if mortality and transfer out was not reported. No study reported on all outcomes and on predictors influencing these outcomes, which made it impossible to assess exactly what happened to these children and why they were lost from follow-up.

Presentation for HIV testing and treatment at a late stage of disease has been shown to increase mortality both before and after ART initiation [22]–[24]. It also increases the risk of developing infectious and non-infectious diseases in HIV-infected children [25]. In this study, we found that the large majority of children were eligible for ART at first presentation. This finding reflects the general failure of health systems to diagnose paediatric HIV-infections and enrol these patients into HIV care early in the course of the disease. Limited capacity to perform PCR testing in infants and to retrieve test results remain important barriers to the success of many ART programmes. Also the lack of integration of PMTCT with paediatric and maternal HIV care programmes [26], stigma, and other socio-cultural barriers are major problems. In a previous meta-analysis, we found that 64% of HIV-exposed infants received early infant diagnosis by PCR at around 6 weeks, and 55% were tested between 12–18 months [27].

Detailed reasons for poor uptake of HIV testing and treatment in low income settings remain poorly understood. No studies included in this review differentiated loss to follow-up in children tested by PCR versus those tested with rapid tests. The feasibility and effect of decentralization of paediatric HIV care could also not be assessed. Since studies evaluating PMTCT programme outcomes were excluded from the analysis, one of the reasons for the high proportion of children with advanced clinical disease at presentation could be that the majority of these children were not included in ART programmes after birth.

Several studies, including two systematic reviews [4], [28], have shown that about one third of adult patients who meet ART eligibility criteria never started ART. In the present study we found that the situation is more encouraging for children since 40% to 99% of treatment eligible children started ART. Table 2 compares the present review with the two previous systematic reviews in adults. Most importantly, more treatment-eligible children than adults start ART. In Rosen *et al* [4], fewer patients were assessed for eligibility but the definition of this second step differed slightly.

**Table 2:** Comparison of different systematic reviews about linkage to care in adults and children.

	Present study	Rosen et al [4]	Mugglin et al [28]
Population	Children	Adults	Adults
**Study location**	Sub-Saharan Africa, India and Cambodia	Sub-Saharan Africa	Sub-Saharan Africa
**Separation into mortality, loss to follow-up and transfer out**	Yes	No	Yes
**Assessed predictors for loss to follow-up**	Yes	No	Yes
**Assessed first CD4 cell count/percentage**	Yes	No	Yes(only absolute CD4 cell count)
**Number of studies**	10	28	29
**Total number of patients analyzed**	10,741	66,926	148,912
**Period of review**	January 2002 to August 2011	All up to April 2011	January 2002 to August 2011
**Percent assessed for eligibility** * **#**	range: 78.0 - 97.0%	59% (35 - 88%)	78% (71 - 84%)
**Percent eligible starting ART** *	range: 39.5 - 99.4%	68% (14 - 84%)	63% (55.71%)

* Percentages with 95% confidence intervals are shown if not stated otherwise

# Rosen: staged and referred for ART or pre-ART care

Mugglin: Provided CD4 sample irrespective or referral to ART or pre-ART care

Not surprisingly low CD4 cell counts and advanced clinical stage of disease were important predictors for starting ART. However, the high proportion of children starting ART in advanced clinical stage could also mean that these children are more likely to access and remain in HIV care whereas healthier children might not be linked to ART programmes or may drop out during follow-up. In rural areas time from diagnosis to ART initiation was longer if the distance to the clinic increased [15]. Structural barriers for retention in HIV care have been described in adults [4] and the same factors also influence clinical outcomes in children.

Our findings underline the need to improve linkage to care and access to ART for HIV-infected children in low-income countries. A wide range of measures and interventions have been proposed to improve clinical outcomes of these patients. These include more widely available point-of-care CD4 cell count testing [5], [6] and removal of barriers to ART in specific populations. The WHO 2010 guidelines that recommend ART for all children <2 years old, regardless of CD4 cell count/percentage [21] are one example. Other approaches to improve retention in PMTCT programmes (e.g. staff training and active defaulter tracing systems [29], [30]) and access to general health care programmes, such as family-centred models [31] have also been proposed. In order to increase the coverage of ART, especially in remote rural settings, HIV testing and care have to be decentralized and brought to the communities [32]. This is particularly important for PCR diagnosis in infants <18 months old.

In conclusion, this systematic review shows that the large majority of children accessing HIV care meet ART eligibility criteria, suggesting that efforts should be made to link children to HIV testing and ART programmes at earlier stages. Pre-ART mortality and loss to follow-up remain important barriers to the improvement of ART coverage in HIV infected children in resource-limited settings. Importantly, data on clinical outcomes and predictors of retention in care during the pre-ART time period are scarce. Future studies should document mortality, loss to follow-up and transfer-out for all pre-ART time periods. HIV testing by PCR in children less than 18 months should be distinguished from other HIV tests. Assessment of eligibility criteria for starting ART should be separated into immunological (absolute and percentage CD4) and clinical criteria and stratified by age. Children lost to programme should be traced and reasons for attrition recorded. Finally, future studies should specifically examine whether universal ART for all children less than two years (irrespective of CD4 cell determination) decreases pre-ART loss to programme in these children.

## Supporting Information

Appendix S1 **Search Terms of Electronic Databases.**
(DOCX)

Table S1 **Mortality, loss to follow-up (LTFU) and transfer out before start of antiretroviral therapy in studies included in the systematic review.** Rates per 100 person-years (pyrs) are given if reported in the study. Percentages refer to the proportion of patients enrolled in care, irrespective of the follow-up time.
(DOCX)

Diagram S1 **PRISMA Flow Diagram.**
(DOC)

Checklist S1 **PRISMA Checklist.**
(DOC)
