# Supplementary material for: Retention in Care of HIV-Infected Children from HIV Test to Start of Antiretroviral Therapy: Systematic Review
Source: PLoS One. 2013 Feb 20;8(2):e56446. doi: 10.1371/journal.pone.0056446 (PMC3577897; doi:10.1371/journal.pone.0056446)
Supplement: Appendix S1 — Search Terms of Electronic Databases. (DOCX) [file pone.0056446.s001.docx]

**Appendix**

**Search Terms of Electronic Databases**

Literature Search: conducted 09.08.2011

Pubmed:

("antiretroviral therapy"[All Fields] OR "anti-retroviral agents/therapeutic use"[Mesh Terms] OR "anti HIV agents"[MeSH Terms] OR "antiretroviral therapy, highly active"[MeSH Terms]) AND ("treatment initiation"[All Fields] OR "pre treatment"[All Fields] OR "prior to treatment"[All Fields] OR "linkage to care"[All Fields] OR "pre-ART"[All Fields] OR eligib* OR "waiting"[All Fields] OR ("lost to follow-up"[All Fields] OR "loss to follow-up"[All Fields] OR ("losses"[All Fields] AND "follow up"[All Fields]))) NOT ("europe"[MeSH Terms] OR "australia"[MeSH Terms] OR "north america"[MeSH Terms]) AND (English[lang] AND ("2002"[PDAT] : "2011"[PDAT]))

Embase:

('antiretroviral therapy' OR (untreated AND 'hiv'/exp) OR 'human immunodeficiency virus infection'/exp/mj OR 'antiretrovirus agent'/exp/mj OR ('human immunodeficiency virus'/exp/mj AND art) OR (arv AND 'human immunodeficiency virus'/exp/mj) OR 'anti human immunodeficiency virus agent'/exp/mj OR 'highly active antiretroviral therapy'/exp/mj) AND ('linkage to treatment' OR 'linkage to care' OR 'lost to care' OR 'loss to care' OR ('losses' AND 'care') OR 'waiting' OR ‘not on ART’ OR ‘retention in care’ OR 'prior to treatment' OR 'pre treatment' OR 'lost to follow-up' OR 'loss to follow-up' OR ('losses' AND 'follow-up'/exp) OR 'pre-ART') AND [humans]/lim AND [english]/lim NOT ('europe'/exp OR 'australia'/exp OR 'north america'/exp) AND [2002-2011]/py
