# Supplementary material for: Retention in Care of HIV-Infected Children from HIV Test to Start of Antiretroviral Therapy: Systematic Review
Source: PLoS One. 2013 Feb 20;8(2):e56446. doi: 10.1371/journal.pone.0056446 (PMC3577897; doi:10.1371/journal.pone.0056446)
Supplement: Table S1 — Mortality, loss to follow-up (LTFU) and transfer out before start of antiretroviral therapy in studies included in the systematic review. Rates per 100 person-years (pyrs) are given if reported in the study. Percentages refer to the proportion of patients enrolled in care, irrespective of the follow-up time. (DOCX) [file pone.0056446.s002.docx]

**Table S1**

| **Study** | **Mortality** | **LTFU** | **Transfer out** |
| --- | --- | --- | --- |
| **Anaky 2010 [11]** | 13.0 (per 100 pyrs) | 50.3 (per 100 pyrs) | 2.9 (per 100 pyrs) |
| **Berhan 2011 [10]** | n/r | n/r | n/r |
| **Edmonds 2011 [12]** | 6.0 (per 100 pyrs) | n/r | n/r |
| **Feucht 2007 [19]** | n/r | n/r | n/r |
| **Leyenaar 2010 [13]** | 13.0% | 6.2% | 3.6% |
| **McGuire 2010 [14]** | 3.2% | 22.3% | 4.6% |
| **Nyandiko 2009 [17]** | n/r | 37% | n/r |
| **Raguenaud 2009 [16]** | 5.7% | 8.6% | 3.4% |
| **Seth 2011 [18]** | 45.8% | 0.0% | n/r |
| **Sutcliffe 2010 [15]** | 1.8% | 14.1% | 1.2% |

n/r, not reported

Numbers refer to the reference list of the main text
