# Supplementary material for: Retention in Care of HIV-Infected Children from HIV Test to Start of Antiretroviral Therapy: Systematic Review
Source: PLoS One. 2013 Feb 20;8(2):e56446. doi: 10.1371/journal.pone.0056446 (PMC3577897; doi:10.1371/journal.pone.0056446)
Supplement: Diagram S1 — PRISMA Flow Diagram. (DOC) [file pone.0056446.s003.doc]

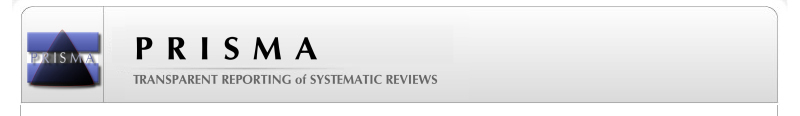
**PRISMA 2009 Flow Diagram**

**Screening**

**Included**

**Eligibility**

**Identification**

Records identified through database searching
(n = 2122 )

Additional records identified through other sources
(n = 0 )

Records after duplicates removed
(n = 1656 )

Records screened
(n = 1656 )

Records excluded
(n = 1575 )

Full-text articles assessed for eligibility
(n = 81 )

Full-text articles excluded, with reasons
(n = 71 )

Studies included in qualitative synthesis
(n = 10 )

Studies included in quantitative synthesis (meta-analysis)
(n = 6 )
